# Prevalence of *Salmonella* Isolates from Chicken and Pig Slaughterhouses and Emergence of Ciprofloxacin and Cefotaxime Co-Resistant *S*. *enterica* Serovar Indiana in Henan, China

**DOI:** 10.1371/journal.pone.0144532

**Published:** 2015-12-09

**Authors:** Li Bai, Ruiting Lan, Xiuli Zhang, Shenghui Cui, Jin Xu, Yunchang Guo, Fengqin Li, Ding Zhang

**Affiliations:** 1 Key Laboratory of Food Safety Risk Assessment, Ministry of Health, China National Center for food safety Risk Assessment, Beijing, China; 2 School of Biotechnology and Biomolecular Sciences, University of New South Wales, Sydney, New South Wales, Australia; 3 Henan Center for Disease Control and Prevention, Henan, China; 4 Department of Food Science, National Institutes for Food and Drug Control, Beijing, People’s Republic of China; California Northstate University College of Medicine, UNITED STATES

## Abstract

The prevalence of *Salmonella* from chicken and pig slaughterhouses in Henan, China and antimicrobial susceptibility of these isolates to antibiotics was determined. From 283 chicken samples and 240 pig samples collected, 128 and 70 *Salmonella* isolates were recovered with an isolation rate of 45.2 and 29.2% respectively. The predominant serovars in chicken samples were *S*. *enterica* serovar Enteritidis, *S*. *enterica* serovar Hadar and *S*. *enterica* serovar Indiana, while those in pig samples were *S*. *enterica* serovar Typhimurium, *S*. *enterica* serovar Derby and *S*. *enterica* serovar Enteritidis. Resistance to ciprofloxacin was 8.6 and 10.0% for isolates from chickens and pigs respectively, whereas resistance to cefotaxime was 5.5 and 8.6%, respectively. Multidrug resistance (resistance to three or more classes of antimicrobial agent) was markedly higher in pig isolates (57.1%) than in chicken isolates (39.8%). Of particular concern was the detection of ciprofloxacin and cefotaxime co-resistant *S*. *enterica* serovar Indiana isolates, which pose risk to public health. All 16 *S*. *enterica* serovar Indiana isolates detected were resistant to ciprofloxacin, among which 11 were co-resistant to cefotaxime. The *S*. *enterica* serovar Indiana isolates accumulated point mutations in quinolone resistance determination regions of *gyrA* (S83F/D87G or S83F/D87N) and *parC* (T57S/S80R). Two plasmid mediated quinolone resistant determinants were found with *aac (6')-Ib-cr* and *oqxAB* in 16 and 12 *S*. *enterica* serovar Indiana isolates respectively. Cefotaxime-resistance of *S*. *enterica* serovar Indiana was associated with the acquisition of a *bla*_CTX-M-65_ gene. The potential risk of ciprofloxacin and cefotaxime co-resistant *S*. *enterica* serovar Indiana infection is a significant concern due to limited alternative treatment options. Reduction of *Salmonella* in chicken and pig slaughterhouses, in particular, ciprofloxacin and cefotaxime co-resistant *S*. *enterica* serovar Indiana will be an important measure to reduce the public health burden of *Salmonella* infections.

## Introduction

Foodborne salmonellosis is an important public health problem worldwide [1]. There is an estimated 94 million annual cases of non-typhoidal *Salmonella* infections with 155,000 deaths and 85% of these cases are foodborne [2]. Salmonellosis is also one of the main causes of morbidity in China, accounting for 75% (30 million cases) of the foodborne diseases [3]. Children younger than 5-years were the group most affected by *Salmonella*-related infection in several cities in China [4,5,6]. Chickens and pigs are the main reservoirs of human foodborne non-typhoidal *Salmonella* infections. Epidemiological investigations have shown that the contaminated raw or undercooked chicken- and pig-meat was the primary vehicle for transmission to humans [7,8]. Fluoroquinolones and extended-spectrum cephalosporins are the most frequently used antimicrobial agents for treating invasive salmonellosis in humans, especially children and the elderly [1,5]. On the other hand, antimicrobials are also used for animal growth promotion and this application can select for and disseminate antimicrobial-resistant *Salmonella*. Multidrug-resistant (MDR) *Salmonella* strains that emerged in animal populations may pass from food animals to humans *via* the food chain [1]. Recent studies have shown that food-producing animals and retail food products might be important reservoirs of fluoroquinolone and/or extended-spectrum cephalosporin-resistant *Salmonella* which have already been reported in many countries [5,9]. *Salmonella enterica* serovar Indiana (*S*. *enterica* serovar Indiana) is one of the most frequently isolated *Salmonella* serovars from many sources [10,11]. And more importantly, the ciprofloxacin and ceftriaxone co-resistant *S*. *enterica* serovar Indiana have been isolated from retail chickens in Beijing and sick animals (ducks and pigs) in Gangdong province, China [10,12].

The prevalence of these co-resistant isolates could be amplified through cross-contamination during the slaughtering process [13]. However, limited data are available for the distribution and characteristics of ciprofloxacin and cefotaxime co-resistant *Salmonella* isolates from chicken and pig slaughterhouses.

In *Salmonella*, ciprofloxacin resistance is mainly attributed to mutations in *gyrA* and *parC* genes [14]. Plasmid-mediated quinolone resistance (PMQR) determinants such as *qnrA*, *qnrB*, *qnrD*, *qnrS*, and *oqxAB* may also contribute to ciprofloxacin resistance [12,15]. Resistance to cefotaxime or other extended spectrum beta-lactams is usually due to intracellular production of extended spectrum β-lactamases (ESBLs). The most commonly found ESBL types in Asia are the CTX-M group, which are usually located on transmissible plasmids that tend to disseminate among members of *Enterobacteriacea* [16]. In this study, *Salmonella* isolated from chicken and pig slaughterhouses were tested for their susceptibility to antimicrobial compounds commonly used in human medicine. The genetic basis of resistance to ciprofloxacin and cefotaxime resistance and the genetic relationships among these isolates were also determined.

## Materials and Methods

### Sample collection and *Salmonella* serotyping

From February to December in 2011, 283 whole chicken carcasses post cooling bath samples from chicken slaughterhouses with processing capacity of 10,000 ~50,000 chickens per day were collected from 2 cities (Hebi and Zhoukou) in Henan province, China. The chicken slaughterhouses were Qixian TIYA and Tangying YUDA in Hebi and Zhoukou TIYA and Shaizai TIYA in Zhoukou. For pig samples, 120 surface swabs of four sites of pig carcasses after evisceration and before chilling and 120 ileocaecal lymph nodes without fat or connective tissue samples from pig slaughterhouses with processing capacity of 2,000~3,000 pigs per day were collected from 2 cities (Hebi and Luohe) in Henan province, China. The pig slaughterhouses were Chengsheng and Xihui in Hebi and Luohe respectively. Both the chicken and pig slaughterhouses were the largest-scale slaughtering and process lines in the regions sampled. No more than five samples were collected from the same slaughterhouse *per* visit during the study. Each sample was put into an aseptic plastic bag, marked and shipped to the laboratory within 24 h on ice. These chickens and pigs were killed as part of the slaughterhouse routine. The whole chicken carcasses and ileocaecal lymph nodes from pigs were purchased during the project.

For whole chicken carcasses samples, each sample was immediately removed from the bag in which it was transported and then placed into a 3500 stomacher bag (Seward, UK) followed by the addition of 500 mL buffered peptone water (BPW; Becton-Dickinson, USA) per kilogram and thoroughly manually massaged for 3–5 minutes to ensure the surface, internal and external of the chicken carcass were fully in contact with the rinse. Then samples were incubated at 37°C for 22–26 h.

Surface swabs for sampling of the pig carcasses were performed after evisceration and before chilling. A surface of about 100 cm^2^ per site was swabbed using one single abrasive sponge for 4 sites (hind limb, abdomen, mid-dorsal region, jowl). The swabs were transferred to 100 mL BPW and incubated at 37°C for 18–20 h. For Lymph nodes samples from pigs, lymph nodes from each pig were removed and pooled, surface de-contaminated before analysis by dipping into 75% (v/v) alcohol and drying in air for 3~5 minutes. Twenty-five grams of lymph nodes were placed into a plastic bag with 225 mL BPW added and mechanistically homogenized by banging with a hammer and then incubated at 37°C for 18–20 h.

For all the samples, 0.5- and 0.1-mL of the pre-enrichment culture were transferred to 10 mL tetrathionate broth (TT; Becton-Dickinson, USA) and Rappaport-Vassiliadis (RV; Becton-Dickinson, USA) broth and incubated at 42±1°C with shaking at 100 rpm for 22–24 h. After selective enrichment, a loopful of TT or RV broth culture was streaked onto xylose lysine tergitol 4 (XLT4; Becton-Dickinson, USA) agar, and incubated at 37±1°C overnight. Three presumptive *Salmonella* colonies on each XLT4 plate were picked and inoculated onto a triple sugar iron slant (Becton-Dickinson, USA) and incubated at 35±1°C for 24 h. Isolates with typical *Salmonella* morphologies were confirmed by amplification of the *invA* gene by PCR [17] and the API 20E biochemical identification system (BioMérieux, Beijing, China). For all confirmed *Salmonella* isolates, serovars were determined by slide agglutination with commercial *Salmonella* antisera (Statens Serum Institute, Denmark) following the Kauffmann-White scheme.

### Antimicrobial susceptibility testing

Antimicrobial susceptibility of all *Salmonella* isolates was determined by the agar dilution method and interpreted according to the Clinical and Laboratory Standards Institute guidelines (CLSI) with the current MIC breakpoints being used [18]: (compounds are abbreviated in parentheses; resistance breakpoints are given in square brackets): ampicillin (AMP [≥32mg/mL]), cefotaxime (CTX [≥4mg/mL]), ceftazidime (CAZ [≥16mg/mL]), chloramphenicol (CHL [≥32mg/mL]), ciprofloxacin (CIP [≥1mg/mL]), gentamicin (GEN [≥16mg/mL]), imipenem (IMP [≥4mg/mL]), nalidixic acid (NAL [≥32mg/mL]), tetracycline (TET [≥16mg/mL]), tigecycline (TGC [≥4mg/mL]) and trimethoprim-sulfamethoxazole (SXT [≥4/76mg/mL]). Multidrug resistance was defined as resistance to three or more classes of antimicrobial agent. Isolates with an MIC 1 mg/L for ceftriaxone or ceftazidime were further screened for extended-spectrum β-lactamase (ESBL) production by determination of synergy between 0.25 and128 mg/L ceftazidime or cefotaxime and 4 mg/L clavulanate. The tigecycline MIC interpretive breakpoint was recommended by the European Committee on Antimicrobial Susceptibility Testing (EUCAST-2012; www.eucast.org). *Escherichia coli* ATCC 25922 and *Klebsiella pneumoniae* ATCC 700603 were used as quality control organisms in antimicrobial susceptibility tests.

### PCR amplification and DNA sequencing of *S*. *enterica* serovar Indiana isolates

The quinolone resistance determination regions (QRDRs) of *gyrA*, *gyrB*, *parC* and *parE* in *Salmonella* isolates were amplified by PCR as described previously [14], sequenced, and then compared to the genes from *Salmonella enterica* serovar Typhimurium LT2 to identify any mutated positions in the target gene. The presence of one or more PMQR genes *qnrA*, *qnrB*, *qnrC*, *qnrD*, *qnrS*, *qepA*, *oqxAB* and *aac(6’)Ib-cr* was determined by PCR and sequencing using primers described previously [12,15]. The genotypes of ESBL-positive isolates were determined by PCR targeting known β-lactamase genes as previously described (*bla*_TEM_, *bla*_SHV_, *bla*_CMY_, *bla*_CTX-M_, and *bla*_OXA_) [12,19]. All the PCR products were either directly sequenced or cloned into pMD18-T plasmid (Takara Biotechnology Cooperation, Dalian, China) for sequence analysis at Takara Biotechnology Cooperation. The sequences obtained were analyzed by Sequencher 4.6 software (Gene Codes Corporation, Ann Arbor, MI, USA). A search for homologous sequences was performed using the BLASTn program at the U.S. National Center for Biotechnology Information website (http://www.ncbi.nlm.nih.gov/BLAST/).

### Pulsed-Field Gel Electrophoresis (PFGE) and Multilocus sequence typing (MLST) of *S*. *enterica* serovar Indiana isolates

Genomic DNA for PFGE was prepared in accordance with the method described previously [20]. Briefly, the genomic DNA of the test strains were digested with 50 U *Xba*I for 2 hours and separated on 1% agarose SeaKem Gold gel with the CHEF DR III system (Bio-Rad, Hercules, California). The PFGE patterns were interpreted with Bionumerics software (Applied Math, Belgium) using Unweighted Pair Group Method with Arithmetic Mean (UPGMA). Bands which were smaller than 20.5 kb were not included in the analysis. Patterns indistinguishable by computer and visual inspection were assigned the same pattern designation, position tolerance of 1%. Clusters were defined as DNA patterns sharing ≥80% similarity (A, B, C).

MLST analysis was conducted by sequencing fragments of seven housekeeping genes (*thrA*, *purE*, *sucA*, *hisD*, *aroC*, *hemD*, *dnaN*) and sequence types (STs) were assigned by comparison to the *S*. *enterica* MLST database (http://mlst.warwick.ac.uk/mlst/dbs/Senterica/documents/primersEnterica_html) [21].

### Plasmid replicon typing and conjugation experiment of *S*. *enterica* serovar Indiana isolates

Plasmids were isolated using Plasmid Plus Midi kit (Qiagen, Germany). Replicon typing of plasmids was performed using a PCR-based method as previously described [22]. Conjugation experiments were performed for all S*almonella* isolates using a sodium azide-resistant *E*. *coli* J53 as recipient strain as previously described [23]. Transconjugants were selected on LB agar plates containing cefotaxime (2 μg/mL) and sodium azide (100 μg/mL).

### Statistical analysis

The Pearson Chi-Square test were used to determine the significant difference (*p* < 0.05) of prevalence of *Salmonella* in chicken and pigs with different characteristics and the rates of resistance between ciprofloxacin and cefotaxime co-resistant *Salmonella* isolates and ciprofloxacin and cefotaxime none-co-resistant *Salmonella* isolates. Statistical software SPSS for Windows (version 17.0, SPSS, Inc., Chicago, IL) was used for descriptive analysis.

## Results

### Prevalence and serotypes of *Salmonella* isolated from chicken and pig samples

Out of the 283 chicken carcasses collected in Hebi (*n* = 141) and Zhoukou (*n* = 142), 128 *Salmonella* isolates were recovered with an isolation rate of 45.2%. The prevalence of *Salmonella* was found to be similar between Hebi (59/141, 41.8%) and Zhoukou (69/142, 48.6%) (χ^2^ = 1.300, *P =* 0.254). Samples collected in the autumn showed the highest contamination rate (77.5%) (Table 1). Serotyping was performed on all isolates. The 128 *Salmonella* isolates belonged to 7 serovars with 10 isolates being defined as untypable. The top three serovars were *S*. *enterica* serovar Enteritidis (*n* = 76, 59.4%), *S*. *enterica* serovar Hadar (*n* = 15, 11.7%) and *S*. *enterica* serovar Indiana (*n* = 11, 8.6%) (Table 2).

**Table 1:** Prevalence of *Salmonella* isolates from chicken slaughterhouses and pig slaughterhouses.

	Chickens						Pigs					
	Hebi		zhoukou		Total		Hebi		Luohe		Total	
Seasons*	No. of samples	No. of positive samples	No. of samples	No. of positive samples	No. of samples	No. of positive samples	No. of samples	No. of positive samples	No. of samples	No. of positive samples	No. of samples	No. of positive samples
Spring	35	15(42.9%)	38	9(23.7%)	73	24(32.9%)	33	8(24.2%)	36	14(38.9%)	69	22(31.9%)
Summer	35	12(34.3%)	35	19(54.3%)	70	31(44.3%)	33	15(45.5%)	24	9(37.5%)	57	24(42.1%)
Autumn	36	25(69.4%)	35	30(85.7%)	71	55(77.5%)	33	7(21.2%)	33	7(21.2%)	66	14(21.2%)
Winter	35	7(20.0%)	34	11(32.4%)	69	18(26.1%)	21	9(42.9%)	27	1(3.7%)	48	10(20.8%)
Total	141	59(41.8%)	142	69(48.6%)	283	128(45.2%)	120	39(32.5%)	120	31(25.8%)	240	70(29.2%)

*Definition of seasons: Spring (March, April, May), Summer (June, July, August), Autumn (September, October, November), Winter (December, January, February)

**Table 2:** Serotyping of *Salmonella* isolates from chicken slaughterhouses and pig slaughterhouses.

Chickens							Pigs						
	No. of isolates							No. of isolates					
Serovar	Hebi(n = 59)		Zhoukou (n = 69)		Total (n = 128)		Serovar	Hebi(n = 39)		Luohe (n = 31)		Total (n = 70)	
Enteritidis	28	(47.5%)	48	(69.6%)	76	(59.4%)	Typhimurium	15	(38.5%)	5	(16.2%)	20	(28.6%)
Hadar	0	(0.0%)	15	(21.7%)	15	(11.7%)	Derby	1	(2.6%)	18	(58.1%)	19	(27.1%)
Indiana	11	(18.6%)	0	(0.0%)	11	(8.6%)	Enteritidis	5	(12.8%)	1	(3.2%)	6	(8.6%)
Typhimurium	7	(11.9%)	0	(0.0%)	7	(5.5%)	Indiana	5	(12.8%)	0	(0.0%)	5	(7.2%)
Infantis	0	(0.0%)	5	(7.2%)	5	(3.9%)	Muenster	5	(12.8%)	0	(0.0%)	5	(7.2%)
Senftenberg	3	(5.1%)	0	(0.0%)	3	(2.3%)	London	4	(10.2%)	0	(0.0%)	4	(5.7%)
Kentucky	1	(1.7%)	0	(0.0%)	1	(0.8%)	Agona	2	(5.1%)	1	(3.2%)	3	(4.3%)
Untyped	9	(15.2%)	1	(1.5%)	10	(7.8%)	Aberdeen	1	(2.6%)	0	(0.0%)	1	(1.4%)
							Meleagridis	0	(0.0%)	1	(3.2%)	1	(1.4%)
							Chester	0	(0.0%)	1	(3.2%)	1	(1.4%)
							Thompson	0	(0.0%)	1	(3.2%)	1	(1.4%)
							Untyped	1	(2.6%)	3	(9.7%)	4	(5.7%)

Out of the 240 samples taken from pigs in Hebi (surface swabs = 60, lymph nodes = 60) and Luohe (surface swabs = 60, lymph nodes = 60), 70 *Salmonella* isolates were recovered with an isolation rate of 29.2%. The prevalence of *Salmonella* was found to be similar between Hebi (39/120, 32.5%) and Luohe (31/120, 25.8%) (χ^2^ = 1.291, *P* = 0.256) (Table 1). However there was a significant difference between surface swabs samples (26/120, 21.7%) and ileocaecal lymph nodes (44/120, 36.7%) (χ^2^ = 6.534, *P* = 0.011). Samples collected in the summer period showed the highest contamination rate (42.1%)(Table 1). Of these 70 pig isolates, 11 serovars were found with 4 isolates being untypable. The top three serovars were *S*. *enterica* serovar Typhimurium (*n* = 20, 28.6%), *S*. *enterica* serovar Derby (*n* = 19, 27.1%), and *S*. *enterica* serovar Enteritidis (*n* = 6, 8.6%) (Table 2).

### Antimicrobial susceptibility testing

Of the 128 chicken isolates studied, 6 were susceptible to all antimicrobial compounds tested and all isolates were susceptible to imipenem and tigecycline. Resistance to nalidixic acid was common (117/128, 91.4%), followed by ampicillin (84/128, 65.6%), tetracycline (60/128, 46.9%). Eleven isolates (8.6%) were resistant to ciprofloxacin, and were all *S*. *enterica* serovar Indiana. Seven isolates (5.5%, 7/128) (*S*. *enterica* serovar Indiana = 6 and *S*. *enterica* serovar Infantis = 1) were ESBL-producers, of which six (85.7%, 6/7) were also resistant to ciprofloxacin. Twenty antimicrobial resistance profiles were identified among the 128 chicken isolates. Multiple drug resistance accounted for 39.8% (51/128) of the chicken isolates. The top 3 antimicrobial resistance profiles of the chicken isolates were AMP-NAL (*n* = 33, 27.0%), NAL alone (*n* = 22, 18.0%), and AMP -NAL-TET (*n* = 19, 15.6%) (Table 3).

**Table 3:** The top 10 antimicrobial resistance profiles of *Salmonella* isolates recovered from chicken slaughterhouses in Henan, China.

Resistant profiles	No. of resistant isolates						
	Enteritidis	Hadar	Typhimurium	Indiana	Infantis	Other Serovars	Total
AMP-CAZ-CHL-CIP-CTX-GEN-NAL-SXT-TET*	0	0	0	6	0	0	6
AMP-CHL-CIP-GEN-NAL-SXT-TET	0	0	0	5	0	0	5
AMP-CHL-GEN-NAL-TET	0	0	6	0	0	0	6
AMP-GEN-NAL-TET	2	2	0	0	0	0	4
AMP-GEN-NAL	6	0	0	0	0	0	6
AMP-NAL-TET	17	2	0	0	0	0	19
NAL-TET	0	8	1	0	0	2	11
AMP-NAL	32	1	0	0	0	0	33
NAL	18	0	0	0	4	0	22
TET	0	1	0	0	0	1	2

^*^AMP, ampicillin; CAZ, ceftazidime; CHL, Chloramphenicol; CIP, ciprofloxacin; CTX, cefotaxime; GEN, gentamicin; SXT, trimethoprim/sulfamethoxazole; TET, tetracycline.

Of the 70 pig isolates, 13 were susceptible to all antimicrobials tested and all isolates were susceptible to imipenem and tigecycline. Resistance to tetracycline was common (47/70, 67.1%) among these isolates, followed by nalidixic acid (45/70, 64.3%), chloramphenicol (42/70, 60.0%). Seven isolates (10.0%, 7/70) of which five and two were *S*. *enterica* serovar Indiana and *S*. *enterica* serovar Typhimurium respectively, were found to be resistant to ciprofloxacin. Six isolates (8.6%, 6/70) (*S*. *enterica* serovar Indiana = 5, *S*. *enterica* serovar Enteritidis = 1) were ESBL-producers and 5 (83.3%, 5/6) of these were also resistant to ciprofloxacin. Eighteen antimicrobial resistance profiles were identified among the 70 pig isolates. Multiple drug resistance accounted for 57.1% (40/70) of the pig collection. The top 3 antimicrobial resistance profiles of pig isolates were CHL-NAL-TET (*n* = 11, 19.3%), AMP-CHL-GEN-NAL-SXT-TET (*n* = 8, 14.0%), and TET alone (*n* = 6, 10.5%) (Table 4).

**Table 4:** The top 10 antimicrobial resistance profiles of *Salmonella* isolates recovered from pig slaughterhouses in Henan, China.

Resistant profiles	No. of resistant isolates						
	Typhimurium	Derby	Indiana	Enteritidis	London	Other Serovars	Total
AMP-CAZ-CHL-CIP-CTX-GEN-NAL-SXT-TET^*^	0	0	5	0	0	0	5
AMP-CHL-GEN-NAL-SXT-TET	8	0	0	0	0	0	8
AMP-CHL-GEN-NAL-TET	5	0	0	0	0	0	5
AMP-CHL-GEN-SXT-TET	0	0	0	0	3	0	3
AMP-CHL-NAL-SXT-TET	1	0	0	0	0	1	2
CHL-NAL-TET	0	11	0	0	0	0	11
AMP-NAL	1	0	0	3	0	1	5
CHL-TET	0	2	0	0	0	0	2
NAL	0	0	0	2	0	0	2
TET	0	0	0	0	0	6	6

^*^AMP, ampicillin; CAZ, ceftazidime; CHL, Chloramphenicol; CIP, ciprofloxacin; CTX, cefotaxime; GEN, gentamicin; SXT, trimethoprim/sulfamethoxazole; TET, tetracycline.

In total, 11 *S*. *enterica* serovar Indiana isolates were identified as being co-resistant to ciprofloxacin and cefotaxime, with 6 and 5 of these being isolated from chickens and pigs, respectively. These isolates were also resistant to 7 other antimicrobial agents (AMP, CAZ, CHL, GEN, NAL, SXT, TET) (Table 5). In contrast, the ciprofloxacin and cefotaxime -co-resistant *Salmonella* isolates alone showed high rate of resistance to most of the antibiotics except nalidixic acid (Table 5).

**Table 5:** Resistance phenotypes of ciprofloxacin and cefotaxime co-resistance *Salmonella* isolates and none ciprofloxacin and cefotaxime co-resistance *Salmonella* isolates from chicken and pig slaughterhouses.

*Antimicrobials*	No. of resistant isolates				
	co-Resistant Isolates (n = 11)		Non co-Resistant Isolates (n = 187)		*p-*Value*
Ampicillin	11	(100.0%)	107	(57.2%)	0.013
Ceftazidime	11	(100.0%)	2	(1.1%)	<0.001
Chloramphenicol	11	(100.0%)	51	(27.3%)	<0.001
Ciprofloxacin	11	(100.0%)	7	(3.7%)	<0.001
Cefotaxime	11	(100.0%)	2	(1.1%)	<0.001
Gentamicin	11	(100.0%)	44	(23.5%)	<0.001
Imipenem	0	(0.0%)	0	(0.0%)	-
Nalidixic acid	11	(100.0%)	151	(80.7%)	0.228
Tetracycline	11	(100.0%)	96	(51.3%)	0.005
Tigecycline	0	(0.0%)	0	(0.0%)	-
Trimethoprim-sulfamethoxazole	11	(100.0%)	22	(11.8%)	<0.001

* *p* value were calculated using the chi-square by SPSS version 17.0.

Nine isolates were ciprofloxacin and cefotaxime non-co-resistant with 7 and 2 *Salmonella* isolates being resistant to ciprofloxacin only and cefotaxime only respectively. The ciprofloxacin resistant only isolates were from two different serovars, and 5 of these were *S*. *enterica* serovar Indiana isolates from chickens along with 2 *S*. *enterica* serovar Typhimurium isolates from pigs. The cefotaxime resistant only isolates were from two different serovars, a *S*. *enterica* serovar Infant isolate from a chicken sample and a *S*. *enterica* serovar Enteritidis isolate from a pig sample.

### Identification of quinolone resistance determinants of *S*. *enterica* serovar Indiana isolates

Sixteen isolates were resistant to ciprofloxacin with minimum inhibitory concentration (MIC) values between 64 and 128 μg /mL. PCR and sequencing was used to identify point mutations in the QRDRs of *gyrA*, g*yrB*, *parC* or p*arE*. in all 16 ciprofloxacin-resistant S. enterica serovar Indiana isolates among which 11 isolates were also resistant to cefotaxime. No mutations were found in *gyrB* nor *parE* in any of the isolates. All possessed 4 point mutations in QRDRs of which 8 isolates had *gyrA* (S83F/D87N), *parC* (T57S/S80R) and 8 isolates had *gyrA* (S83F/D87G), *parC* (T57S/S80R) mutations.

We also determined the carriage of the PMQR determinants and found that all 16 isolates carried *aac(6')-Ib-cr*, among which 12 isolates also carried *oqxAB*. None of the isolates carried *qnrA*, *qnrB*, *qnrC*, *qnrD*, *qnrS* or *qepA*.

### Occurrence of β-lactamase-encoding genes of *S*. *enterica* serovar Indiana isolates

Among the 16 *S*. *enterica* serovar Indiana isolates, 11 carried genes encoding CTX-M-type ESBLs and the subtype of *bla*_CTX-M_ was identified as *bla*_CTX-M-65_ for all 11 isolates. The ESBL genotypes, *bla*_OXA-1_ and *bla*_TEM-1_ were also identified in 16 and 5 isolates respectively.

### PFGE and MLST analysis of *S*. *enterica* serovar Indiana isolates

**Fig 1:**
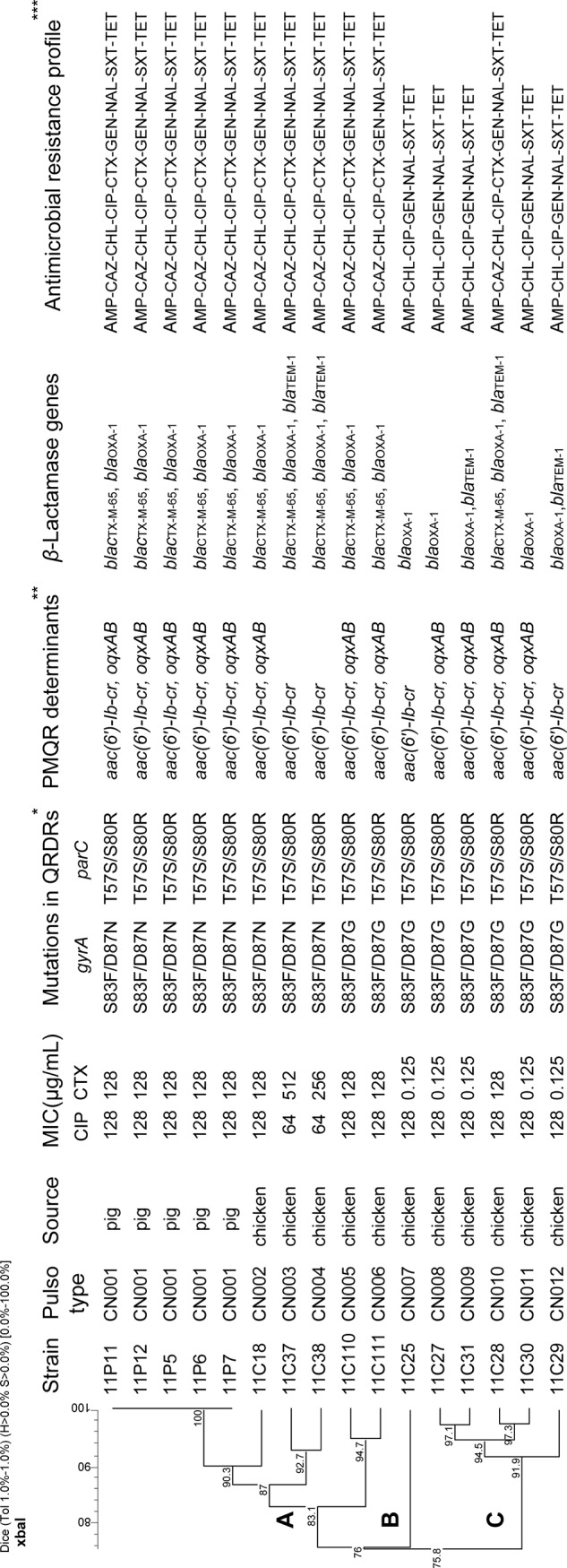
Dendrogram of 16 *S*. *enterica* serovar Indiana isolates constructed based on *Xba*I PFGE patterns. Stains information, pulsotype, mutations in QRDRs, resistance determinants/genes and resistance profiles are shown on the right. * PMQR, Plasmid Mediated Quinolone Resistance. **QRDRs, Quinolone Resistance Determining Regions. ***AMP, ampicillin; CAZ, ceftazidime; CHL, chloramphenicol; CIP, ciprofloxacin; CTX, cefotaxime; GEN, gentamicin; SXT, trimethoprim/sulfamethoxazole; TET, tetracycline.

To further elucidate the genetic relationships of the 16 ciprofloxacin and/or cefotaxime resistant *S*. *enterica* serovar Indiana isolates by MLST and PFGE. All 16 isolates belonged to the same sequence type ST17. By PFGE, the 16 isolates were clustered into 12 pulsotypes. A dendrogram was constructed using these PFGE patterns. Isolates grouped into the same PFGE patterns mostly were recovered from the same regions. The predominant pulsotype was denoted as CN001, which included five isolates from the same pig slaughterhouse in Hebi on the same day (23^rd^, February 2011). The remaining 11 pulsotypes contained a single isolate (Fig 1). Cluster A consisted of mainly ciprofloxacin and cefotaxime co-resistant *Salmonella* isolates of which the isolates from chicken and pig slaughterhouses were different. Cluster C contained only ciprofloxacin resistant *Salmonella* isolates with the exception of isolate 11C28. Those isolates containing point mutations in *gyrA* (S83F/D87N) and *parC* (T57S/S80R) were all grouped together in cluster A whilst those with point mutations in *gyrA* (S83F/D87G) and *parC* (T57S/S80R) were mostly grouped into clusters B and C.

### Plasmid replicon typing and conjugation experiment of ESBL-*S*. *enterica* serovar Indiana isolates

Conjugation experiments were conducted on these isolates using *E*. *coli* J53 as a recipient strain. However, no transconjugants were obtained. Seven isolates contained plasmids belonging to the IncN group and none of the 18 plasmids was detected in the rest of 4 ESBL-*Salmonella* isolates.

## Discussion

In this study, we screened processed chickens and pigs in slaughterhouses collected from 3 districts of Henan province which was one of the largest food-animal processing provinces in China for the presence of *Salmonella*, and further characterized the isolates using serotyping and of antimicrobial susceptibility testing. Specifically the genotypic mechanisms of ciprofloxacin-resistant and cefotaxime-resistant isolates were identified.

There had been a few studies on prevalence of *Salmonella* in poultry in retail markets in China wherein values varied from 38.9 to 65.3% depending on the geographical [24]. Our isolation rate of 45.2% from chickens in slaughterhouses was similar to that of 47.9% reported earlier from retail markets in the same (Henan) province [24], suggesting similar levels of contamination in the entire supply chain. However, our isolation rate was much higher than that (22.1%) from a slaughterhouse in Anhui province reported by Wang *et al*. [11]. The top serovars found in this study were *S*. *enterica* serovar Enteritidis, *S*. *enterica* serovar Indiana and S. *enterica* serovar Typhimurium. Two of the three (*S*. *enterica* serovar Enteritidis, *S*. *enterica* serovar Indiana) were also counted in the top three *Salmonella* serovars (*S*. *enterica* serovar Enteritidis, *S*. *enterica* serovar Hadar and *S*. *enterica* serovar Indiana) in chickens in China as reported by other studies [10,25,26]. These serovars were within the top four serovars (*S*. *enterica* serovar Typhimurium, *S*. *enterica* serovar Enteritidis, *S*. *enterica* serovar Derby, *S*. *enterica* serovar Indiana) isolated from patients in Henan from 2006 to 2007 [27], consistent with poultry as a major source of *Salmonella* infection in humans.

The isolation of *Salmonella* from pigs was much lower and the prevalent serovars were also different. The isolation rate of 29.2% was higher than the isolation rate from slaughterhouses reported by the EFSA (10.3%) [28] and Li *et al*. (12.0%) [29], but similar to that from pork in retail market (31%) reported by Yang *et al*. [30]. The differences were likely due to sources or regional difference. The common serovars found in this study (*S*. *enterica* serovar Typhimurium, *S*. *enterica* serovar Derby and *S*. *enterica* serovar Enteritidis) were also reported to be common in pigs by the EFSA [28].

*Salmonella* is still the leading cause of hospitalization in USA [31] and MDR *Salmonella* has become a significant public health concern worldwide [32]. Our data showed that nearly half of the isolates (46.0%) from Henan province were multidrug resistant which was the same as that (46.0%) from patients in Guangdong province [4] and a somewhat higher than that (34.9%) in Beijing [10] but was sharply lower than that (93%) in Anhui province [11]. The high occurrence of multidrug resistant *Salmonella* in slaughterhouses indicates that cross-contamination in the subsequent retail process poses serious risk to public health. With the large-scale use, and often abuse, of antimicrobials over time, fluoroquinolone and/or extended-spectrum cephalosporin resistant *Salmonella* isolates have been detected from patients in numerous locations with variable prevalence in China, such as 6.7% in Gangdong province [4], 12.2% in Wuhan [5], 17.2% in Shanghai [6] and 26.9% in Henan province [27]. Importantly, 10.1% *Salmonella* isolates exhibited resistance to ciprofloxacin or third generation cephalosporin in this study. Fluoroquinolone resistance in *Salmonella* is a unique problem in China and other Asian countries where there is no strict control on its availability [5,33], in contrast to countries with strict antimicrobial controls, such as the United States, where less than 3% of *Salmonella* isolates were considered resistant to ciprofloxacin [34] and in Belgium, wherein resistance to ciprofloxacxin was undetectable in isolates from both healthy pigs and chickens [35].

In this study, we found 11 *Salmonella* isolates that were co-resistant to ciprofloxacin and cefotaxime and all of which were *S*. *enterica* serovar Indiana. The appearance of ciprofloxacin and cefotaxime co-resistant *S*. *enterica* serovar Indiana is alarming. It seems that the co-resistance only developed recently since the study by Xia *et al*. in 2009 [27] found that *S*. *enterica* serovar Indiana from Henan province were susceptible to cefotaxime [27]. The emergence of cefotaxime resistant *S*. *enterica* serovar Indiana could either be due to acquisition of cefotaxime resistance genes by local strains or through spread from other regions where ciprofloxacin and cefotaxime co-resistant *S*. *enterica* serovar Indiana has been reported such as Beijing [10] or by cross-genus spread from ciprofloxacin and cefotaxime co-resistant *E*. *coli* which has been reported in Gangdong province, China [36].

Two PMQR determinants were identified in our *S*. *enterica* serovar Indiana isolates with*aac(6')-Ib-cr* present in all 16 isolates and *oqxAB* present in 12 isolates. The distribution of the *oqxAB*-positive *S*. *enterica* serovar Indiana isolates on the PFGE dendrogram (Fig 1) suggests that it was acquired independently 3 times. OqxAB, a novel efflux pump, that mediates resistance to olaquindox and also extrudes antibiotics such as chloramphenicol and fluoroquinolones, broadening the resistance phenotype to these latter antibiotics [37]. Olaquindox, a quinoxaline derivative antibiotic, is as a growth promoter that has been in use in animals for decades in China. The latter feature is suggestive of a constant selective pressure for the acquisition and dissemination of *oqxAB* [12,38]. The co-existence of PMQR genes, *aac(6')-Ib-cr* and *oqxAB*, had been detected previously in both human and animal *Salmonella* isolates in China [12]. Although PMQR confers only low-level quinolone resistance, it can facilitate the emergence of high-level resistance via mutations in a topoisomerase gene *gyrA*, *parC* or both [39]. This may be also the case for the 16 *S*. *enterica* serovar Indiana isolates in this study, PMQR combined with multiple mutations in QRDRs [*gyrA* (S83F/D87N) or *gyrA* (S83F/D87G), *parC* (T57S/S80R)] resulted in high-level resistance to ciprofloxacin (MIC, ≥ 64μg/mL).

Uniquely in China, our study found that *bla*_CTX-M-65_ was carried by the 11 cefotaxime resistant *S*. *enterica* serovar Indiana isolates. In contrast *bla*_CTX-M-14_ and *bla*_CTX-M-27_ have been found in *S*. *enterica* serovar Indiana isolates from chickens and pigs in Gangdong [12] and *bla*_CTX-M-24_ was found in *S*. *enterica* serovar Indiana isolates from chickens in Shandong [40]. These *bla*_CTX-M_ genes are usually located on conjugative plasmids [16]. The *bla*_CTX-M-24_ found in *S*. *enterica* serovar Indiana by Lai *et al*. was shown to be encoded on a unique class 1 integron with multiple resistance genes co-located on a IncHI2 plasmid [40] whilst *bla*_CTX-M-14_ and *bla*_CTX-M-27_ were found in *S*. *enterica* serovar Indiana by Jiang *et al*. and was shown to be encoded on IncHI2 and/or IncN plasmid [12]. Similarly *bla*_CTX-M-24_,*bla*_CTX-M-27_ and *bla*_CTX-M-65_ have been reported in *E*. *coli* from both animals and humans in China [36,41]. It is possible that these *bla*_CTX-M_ genes were acquired by *Salmonella* from *E*. *coli*.

The genetic relationship of the *S*. *enterica* serovar Indiana isolates based on PFGE revealed that all except one ciprofloxacin and cefotaxime co-resistant isolate were grouped together in one cluster, denoted as cluster A, suggesting a single origin. The pig isolates were identical and belonged to this cluster, indicating likely the possible spread from chickens to pigs. The ciprofloxacin and cefotaxime co-resistant isolate located outside the cluster was likely to have acquired the *bla*_CTX-M-65_ gene independently. The isolate also carried a different *gyrA* mutation (S83F/D87G), different from those of the isolates in cluster A, lending further support to its independent origin. Therefore both clonal spread and independent acquisition of cefotaxime resistance may have acted to contribute to the emergence and spread of ciprofloxacin and cefotaxime co-resistant *S*. *enterica* serovar Indiana in Henan province.

Multidrug resistant *S*. *enterica* serovar Indiana has already been reported in human infections in China [6,27]. All *S*. *enterica* serovar Indiana isolates from human infections in Henan were resistant to ciprofloxacin but susceptible to cefotaxime [27]. It is inevitable that the ciprofloxacin and cefotaxime co-resistant *S*. *enterica* serovar Indiana emerged in Henan will transmit to humans, posing a significant threat to public health. The co-resistance limits the optimal treatment alternatives of salmonellosis in humans.

## Conclusion

The prevalence of *Salmonella* in the chicken and pig slaughterhouses in Henan was found to be high, and represented by a small number of different serovars such as *S*. *enterica* serovar Enteritidis, *S*. *enterica* serovar Hadar and *S*. *enterica* serovar Indiana in chickens and *S*. *enterica* serovar Typhimurium, *S*. *enterica* serovar Derby and *S*. *enterica* serovar Enteritidis in pigs. This finding highlights the fact that food-producing animals at processing plants are a significant reservoir of antimicrobial-resistant *Salmonella*. Our data may contribute to the development of interventions at the processing level to interrupt the transmission of *Salmonella* to humans. Ciprofloxacin and cefotaxime co-resistant *S*. *enterica* serovar Indiana possibly emerged only recently through acquisition of *bla*_CTX-M-65_. The potential risk of ciprofloxacin and cefotaxime co-resistant *S*. *enterica* serovar Indiana infections in humans requires urgent attention. The emergence of ciprofloxacin and cefotaxime co-resistance also underscores the importance of strict control on the use of antibiotics in animal production to prevent the emergence of antibiotic resistance in *Salmonella*.
